# Correction: Hybrid thiazolyl–benzylidene–phenol metal complexes as novel chemotherapeutic agents with anti-topoisomerase I activity in human breast carcinoma: synthesis, *in vitro* and *in silico* studies

**DOI:** 10.1039/d5ra90086g

**Published:** 2025-07-14

**Authors:** Ahmed M. Alharbi, Mona Katary, Khulud M. Alshehri, Basim H. Asghar, Mohmed M. Omran, Reda F. M. Elshaarawy, Amira Mili, Hani S. Hafez, Rozan Zakaria

**Affiliations:** a Department of Chemistry, Faculty of Sciences, Umm Al-Qura University Makkah Saudi Arabia amaharbi@uqu.edu.sa; b Chemistry Department, Faculty of Science, Port-Said University Port-Said Egypt monakatary@gmail.com; c Department of Basic Sciences, Faculty of Applied Health Sciences Technology, Horus University Costal Road New Damietta Egypt mkatary@horus.edu.eg; d Department of Biology, Al-Baha University Saudi Arabia kalshehri@bu.edu.sa; e Department of Chemistry, Faculty of Sciences, Umm Al-Qura University Makkah Saudi Arabia bhasghar@uqu.edu.sa; f Chemistry Department, Faculty of Science, Helwan University Helwan Egypt Mohmed.Omran@gmail.com; g Department of Chemistry, Faculty of Science, Suez University 43533 Suez Egypt reda.elshaarawy@suezuniv.edu.eg; h Institut für Anorganische Chemie und Strukturchemie, Heinrich-Heine Universität Düsseldorf Düsseldorf Germany; i Department of Biology, Al-Baha University Al Aqiq Saudi Arabia ahajsalah@bu.edu.sa; j Department of Zoology, Faculty of Science, Suez University 43533 Suez Egypt hani.hafez@suezuniv.edu.eg; k Chemistry Department, Faculty of Science, Port-Said University Port-Said Egypt rozan.zakaria@sci.psu.edu.eg

## Abstract

Correction for ‘Hybrid thiazolyl–benzylidene–phenol metal complexes as novel chemotherapeutic agents with anti-topoisomerase I activity in human breast carcinoma: synthesis, *in vitro* and *in silico* studies’ by Ahmed M. Alharbi *et al.*, *RSC Adv.*, 2025, **15**, 20552–20569, https://doi.org/10.1039/D5RA00889A.

Dr Rozan Zakaria, one of the authors of this manuscript, was incorrectly named as Rozan Zakrya in the original article. The authors regret the misspelling of the email address of one of the authors, Rozan Zakaria (rozan.zakaria@sci.psu.edu.eg), in the published article.

The authors regret an error in affiliation *c* of the original article. The correct list of authors, emails and affiliations are as displayed in this notice.

The Royal Society of Chemistry apologises for these errors and any consequent inconvenience to authors and readers.

